# *In Vitro* Antiviral Activity and Resistance Profile of the Next-Generation Hepatitis C Virus NS5A Inhibitor Pibrentasvir

**DOI:** 10.1128/AAC.02558-16

**Published:** 2017-04-24

**Authors:** Teresa I. Ng, Preethi Krishnan, Tami Pilot-Matias, Warren Kati, Gretja Schnell, Jill Beyer, Thomas Reisch, Liangjun Lu, Tatyana Dekhtyar, Michelle Irvin, Rakesh Tripathi, Clarence Maring, John T. Randolph, Rolf Wagner, Christine Collins

**Affiliations:** AbbVie, Inc., North Chicago, Illinois, USA

**Keywords:** pibrentasvir, NS5A inhibitor, HCV, resistance, ABT-530, antiviral activity

## Abstract

Pibrentasvir (ABT-530) is a novel and pan-genotypic hepatitis C virus (HCV) NS5A inhibitor with 50% effective concentration (EC_50_) values ranging from 1.4 to 5.0 pM against HCV replicons containing NS5A from genotypes 1 to 6. Pibrentasvir demonstrated similar activity against a panel of chimeric replicons containing HCV NS5A of genotypes 1 to 6 from clinical samples. Resistance selection studies were conducted using HCV replicon cells with NS5A from genotype 1a, 1b, 2a, 2b, 3a, 4a, 5a, or 6a at a concentration of pibrentasvir that was 10- or 100-fold over its EC_50_ for the respective replicon. With pibrentasvir at 10-fold over the respective EC_50_, only a small number of colonies (0.00015 to 0.0065% of input cells) with resistance-associated amino acid substitutions were selected in replicons containing genotype 1a, 2a, or 3a NS5A, and no viable colonies were selected in replicons containing NS5A from other genotypes. With pibrentasvir at 100-fold over the respective EC_50_, very few colonies (0.0002% of input cells) were selected by pibrentasvir in genotype 1a replicon cells while no colonies were selected in other replicons. Pibrentasvir is active against common resistance-conferring substitutions in HCV genotypes 1 to 6 that were identified for other NS5A inhibitors, including those at key amino acid positions 28, 30, 31, or 93. The combination of pibrentasvir with HCV inhibitors of other classes produced synergistic inhibition of HCV replication. In summary, pibrentasvir is a next-generation HCV NS5A inhibitor with potent and pan-genotypic activity, and it maintains activity against common amino acid substitutions of HCV genotypes 1 to 6 that are known to confer resistance to currently approved NS5A inhibitors.

## INTRODUCTION

Hepatitis C virus (HCV) infects approximately 3 million individuals in the United States and 180 million people worldwide (1, 2). Seven HCV genotypes and >60 subtypes have been identified, each with a different geographic distribution (1–3). Genotype 1 HCV accounts for approximately 45% of all infections worldwide, while genotype 3 is next in prevalence (1, 2). A high prevalence of genotype 3 has been reported in Australia, South Asia, and a few European countries, while genotype 2 is more common in East and Southeast Asia (1, 2). Genotype 4 is prevalent in Egypt and the Middle East (2, 4). Genotypes 5 and 6 are found in South Africa and Southeast Asia, respectively. In the United States, genotype 1 accounts for 70% to 75% of all HCV infections. Patients chronically infected with HCV are at high risk of developing cirrhosis and hepatocellular carcinoma (5). Successful treatment of HCV infection has been shown to significantly reduce the risk of liver disease progression and the development of hepatocellular carcinoma (6).

Interferon alpha (IFN-α)-based regimens used to be the standard of care for the treatment of chronic HCV infection, but the recent discovery and approval of direct-acting antiviral agents (DAAs) have revolutionized HCV treatment options (7). Compared to IFN-α-based regimens, DAA-containing regimens are associated with significant improvement in sustained virologic response (SVR) rate and safety profile. HCV DAAs approved since 2011 include HCV NS3/4A protease inhibitors (PIs), NS5A inhibitors, and nucleos(t)ide and nonnucleoside NS5B polymerase inhibitors (7–9). While the success rate of HCV therapy has improved with combination regimens containing DAAs, most of these regimens are not effective for all HCV genotypes; some of them have reduced activity against certain baseline resistance-associated polymorphisms, and DAA-resistant amino acid substitutions can emerge in patients who experience on-treatment virologic breakthrough or posttreatment relapse (8–14). There is a clear unmet medical need for a potent IFN-free regimen with efficacy in patients infected with different HCV genotypes, as well as in patients with virus containing baseline polymorphisms and/or DAA treatment-emergent substitutions.

The HCV NS5A protein plays multiple roles in supporting replication of HCV and is therefore an excellent target for the discovery of anti-HCV therapeutics. The HCV NS5A protein supports the replication of HCV by interacting with viral and cellular proteins to form the HCV replicase complex, the microorganelle that replicates HCV RNA (15). Previous studies also indicate that NS5A plays a critical role in the later stages of the viral life cycle, including the assembly of viral particles into infectious virions (16). Discoveries of a number of NS5A inhibitors with picomolar potency against several HCV genotypes *in vitro* have been reported, and results from studies with first-generation approved HCV NS5A inhibitors, including ombitasvir, daclatasvir, and ledipasvir, validated the clinical efficacy of NS5A inhibitors (17–19). However, all currently approved NS5A inhibitors differ in their antiviral activities against different HCV genotypes and subtypes (20–25).

**FIG 1:**
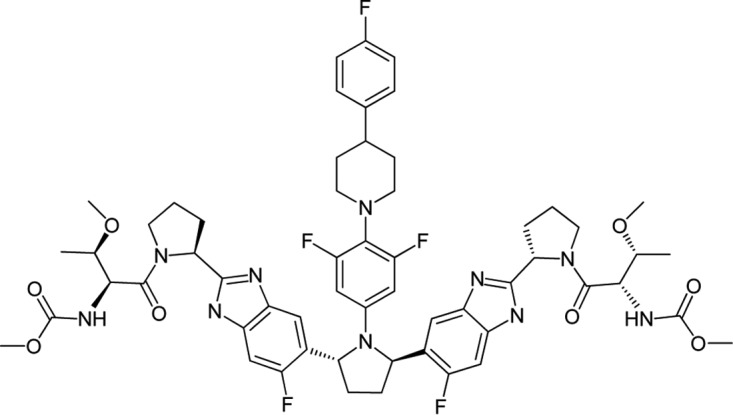
Chemical structure of pibrentasvir.

In this report, we describe the *in vitro* properties of the novel HCV NS5A inhibitor pibrentasvir (ABT-530) (Fig. 1). We evaluated the activity of pibrentasvir in stable HCV replicons containing NS5A from genotypes 1 to 6 and in transiently replicating HCV replicons containing NS5A from HCV-infected patient samples across different genotypes. We also identified and characterized resistance-associated amino acid substitutions selected by pibrentasvir in HCV replicons containing NS5A from genotypes 1 to 6. Furthermore, we tested the activity of pibrentasvir against replicons containing NS5A from genotypes 1 to 6 with amino acid substitutions that confer resistance to other NS5A inhibitors and examined the antiviral effect of the combination of pibrentasvir with HCV inhibitors of other classes.

## RESULTS

### Antiviral activity and therapeutic index of pibrentasvir *in vitro*.

Pibrentasvir demonstrated picomolar and pan-genotypic antiviral activity against a panel of stable replicons with NS5A sequences from HCV genotypes 1 to 6. Pibrentasvir inhibited HCV genotype 1a-H77, 1b-Con1, and 2a-JFH-1 subgenomic replicons with 50% effective concentrations (EC_50_s) of 1.8, 4.3, and 5.0 pM, respectively; EC_50_s ranged between 1.4 and 2.8 pM against chimeric stable replicons containing NS5A derived from HCV genotypes 2a, 2b, 3a, 4a, 5a, and 6a (Table 1). The antiviral activity of pibrentasvir was attenuated 35- to 47-fold in the presence of 40% human plasma through sequestration of compound due to plasma protein binding. The 50% cytotoxic concentration (CC_50_) value of pibrentasvir in an HCV genotype 1a replicon cell line was >32,000,000 pM, producing an *in vitro* therapeutic index that exceeded 10^7^-fold (Table 2). The pibrentasvir CC_50_ values measured in two additional cell lines, HepG2 and MT4, were >10,000,000 pM (Table 2). Pibrentasvir had no measurable antiviral activity against either human immunodeficiency virus type 1 (HIV-1) or hepatitis B virus (HBV) *in vitro* (HIV-1 EC_50_, >900,000 pM; HBV EC_50_, >32,000,000 pM) (Table 1).

**TABLE 1:** Antiviral activity of pibrentasvir *in vitro*

HCV replicon or virus	Pibrentasvir EC_50_ (pM)^*c*^
HCV stable replicons in 0% human plasma^*a*^	
GT1a H77	1.8 ± 0.86
GT1b Con1	4.3 ± 1.7
GT2a JFH-1	5.0 ± 0.60
GT2a^*b*^	2.3 ± 0.65
GT2b	1.9 ± 0.59
GT3a	2.1 ± 0.66
GT4a	1.9 ± 0.61
GT5a	1.4 ± 0.36
GT6a	2.8 ± 0.67
HCV stable replicons in 40% human plasma^*a*^	
GT1a	64 ± 14
GT1b	200 ± 54
HIV-1	>900,000
HBV	>32,000,000

a Both the 0% and 40% human plasma assays also contained 5% fetal bovine serum.

b The genotype 2a (GT2a) replicon contained M31 in NS5A.

c Values are means ± standard deviations. EC_50_, 50% effective concentration.

**TABLE 2:** Cytotoxicity of pibrentasvir *in vitro*

Cell line	Pibrentasvir CC_50_ (pM)^*a*^
Huh-7 (genotype 1a replicon)	>32,000,000
HepG2	>10,000,000
MT4	>10,000,000

a CC_50_, 50% cytotoxic concentration.

To characterize the breadth of coverage of pibrentasvir against HCV clinical samples, the phenotypic susceptibility of a panel of replicons containing NS5A genes from HCV-infected patients was evaluated (Table 3). This panel consisted of 6 to 14 patient samples for each of the 1a, 1b, 2a, 2b, 3a, and 4a genotypes but fewer samples for genotypes 5a, 6a, 6e, and 6p due to limited availability. Some of these samples had amino acid polymorphisms within the NS5A N-terminal region at positions associated with resistance to some NS5A inhibitors (10, 13, 14). For example, methionine (M) and leucine (L) are commonly seen at NS5A amino acid position 31 of genotype 2; genotype 2a and 2b samples with NS5A M31 are less susceptible to daclatasvir, ledipasvir, and elbasvir than those with L31 (23, 26). In the panel of patient samples that we analyzed, 5/6 of the genotype 2a and 4/11 of the genotype 2b samples had M31 in NS5A. In genotype 3a samples, 3/14 and 2/14 samples had A30K and Y93H, respectively, which are associated with resistance to different NS5A inhibitors (21, 22, 25, 27–29). Despite the presence of NS5A polymorphisms in some of these clinical samples, pibrentasvir retained its activity against this panel of replicons, with the median EC_50_s ranging from 0.50 to 2.7 pM (*n* = 64).

**TABLE 3:** Antiviral activity of pibrentasvir against HCV replicons containing NS5A genes from HCV-infected patients

HCV genotype	No. of samples	Pibrentasvir EC_50_ (pM)^*g*^	
		Range	Median (IQR)
1a	11	0.55–1.7	0.89 (0.54)
1b	8	1.4–3.5	2.7 (1.2)
2a^*a*^	6	0.52–1.9	0.93 (0.32)
2b^*b*^	11	1.1–1.9	1.3 (0.27)
3a^*c*^	14	0.47–1.7	0.71 (0.4)
4a^*d*^	8	0.27–1.3	0.50 (0.13)
5a	1	NA^*h*^	1.1
6a^*e*^	3	0.63–1.0	0.74 (0.2)
6e^*f*^	1	NA	0.83
6p	1	NA	0.50

a Five of the genotype 2a samples had M31 in NS5A. One sample contained NS5A from 2a-JFH-1 and had L31.

b Seven of the genotype 2b samples had L31, two had M31, and two had M/L31.

c Three of the genotype 3a samples had A30K, one had A30T, and two had Y93H.

d One of the genotype 4a samples had K24R, one had L28M, one had L30R, and one had P58T.

e Two of genotype 6a samples had F28L.

f The genotype 6e sample had K24R and V28M.

g As determined in transient-transfection assays. IQR, interquartile range.

h NA, not applicable.

### Selection of amino acid substitutions by pibrentasvir in stable replicons of HCV genotypes 1 to 6.

To determine the *in vitro* resistance profile of pibrentasvir, drug-resistant colony selection was conducted with pibrentasvir in HCV replicons containing NS5A from genotype 1a, 1b, 2a, 2b, 3a, 4a, 5a, or 6a. Amino acid substitutions identified in colonies after selection with pibrentasvir treatment are reported in Table 4. For genotype 1a drug-resistant colony selection, 0.0065% or 0.0002% of the input replicon cells survived treatment at a concentration of pibrentasvir that was 10- or 100-fold above its EC_50_, respectively. With pibrentasvir at 10-fold over the EC_50_, the major genotype 1a amino acid substitution selected in NS5A was Y93H, seen in 90% (18/20) of the colonies analyzed after resistance selection. With pibrentasvir at 100-fold over the EC_50_, only four genotype 1a drug-resistant colonies survived out of 2 × 10^6^ input cells, with different amino acid substitutions in NS5A for each colony: Q30D, Q30 deletion, Y93D, and the double substitution H58D+Y93H. In genotype 1b replicon cells, no resistant colonies were selected by pibrentasvir at 10-fold over the EC_50_, and therefore, no selection was performed at higher concentrations.

**TABLE 4:** Selection by pibrentasvir of amino acid substitutions in NS5A from HCV genotypes 1 to 6

HCV genotype^*a*^	Colony survival (%)^*b*^		NS5A amino acid substitution(s)	Prevalence in replicon selection^*c*^		Mean EC_50_ ± SD (pM)	Fold change in EC_50_^*d*^	Replication efficiency (%)^*d*^
	10× EC_50_	100× EC_50_		10× EC_50_	100× EC_50_			
1a^*e*^	0.0065	0.0002	Q30D^*f*^	0/20	1/4^*g*^	68 ± 37	94	50
			Q30 deletion	0/20	1/4^*g*^	2,555 ± 268	3,549	0.5
			Y93D	0/20	1/4^*g*^	NV^*j*^	NV	<0.5
			Y93H	18/20	0/4^*g*^	4.8 ± 1.5	6.7	18
			Y93N	1/20	0/4^*g*^	5.1 ± 2.1	7.1	25
			H58D Y93H	0/20	1/4^*g*^	1,612 ± 272	2,238	13
1b	0	ND	NA	NA^*i*^	NA	NA	NA	
2a^*e*^	0.00015	0	F28S M31I	2/3^*g*^	NA	14,303 ± 2,722	14,448	
			P29S K30G	1/3^*g*^	NA	2.3 ± 0.36	2.3	
2b	0	0	NA	NA	NA	NA	NA	
3a^*e*^	0.0003	0	Y93H	3/3^*h*^	NA	1.5 ± 0.19	2.3	
4a	0	0	NA	NA	NA	NA	NA	
5a	0	0	NA	NA	NA	NA	NA	
6a	0	0	NA	NA	NA	NA	NA	

a Genotype of NS5A in replicon cell lines.

b Calculated as follows: (number of surviving colonies/number of input replicon cells) × 100. ND, not determined; EC_50_, pibrentasvir 50% effective concentration.

c Number of times an amino acid substitution was found/the total number of colonies analyzed.

d Relative to the respective wild-type replicon.

e Pibrentasvir EC_50_s for wild-type replicons in transient-transfection assays were as follows: genotype 1a, 0.72 pM; genotype 2a, 0.99 pM; genotype 3a, 0.65 pM.

f Substitution with double nucleotide changes.

g Denominator indicates the total number of colonies that survived selection out of 2 × 10^6^ input cells.

h Denominator indicates the total number of colonies that survived selection out of 1 × 10^6^ input cells.

i NA, not applicable.

j NV, not available as the EC_50_ could not be determined due to low replication efficiency of the replicon containing the amino acid substitution.

Similar selection studies were performed in cells with HCV chimeric replicons containing NS5A from genotype 2a, 2b, 3a, 4a, 5a, or 6a with pibrentasvir at 10- or 100-fold above its EC_50_s for the respective replicon cells (Table 4). A small number of the input replicon cells containing NS5A from genotype 2a or 3a, 0.00015% or 0.0003%, respectively, survived the selection with 10-fold the pibrentasvir EC_50_. No colonies with resistance-associated substitutions were selected in replicons containing NS5A from genotype 2b, 4a, 5a, and 6a at 10-fold over the respective pibrentasvir EC_50_ concentrations. Selection with 100-fold over the respective pibrentasvir EC_50_ concentrations did not produce any resistant colonies in replicon cells containing NS5A from genotype 2a, 2b, 3a, 4a, 5a, or 6a. Some replicon cells containing genotype 2b, 3a, or 4a NS5A were observed at the completion of 10-fold EC_50_ selection study, but they did not form colonies and were not viable during subsequent passaging; no NS5A amino acid substitutions were detected in these cells.

For drug-resistant colony selection with replicon cells containing genotype 2a NS5A, only three colonies out of 2 × 10^6^ input cells survived the 10-fold EC_50_ selection: two colonies harbored F28S+M31I, and one colony had P29S+K30G. Three replicon colonies containing genotype 3a NS5A survived 10-fold EC_50_ selection out of 1 × 10^6^ input cells; all colonies contained Y93H.

### Activity of pibrentasvir against HCV replicons containing NS5A substitutions selected by pibrentasvir *in vitro*.

Most viruses that emerge upon virologic failure during or after DAA treatment of HCV-infected patients contain amino acid substitutions that confer resistance to the DAAs used for treatment (13, 14). The susceptibility to pibrentasvir of HCV replicons engineered with specific NS5A amino acid substitutions observed during *in vitro* resistance selection with pibrentasvir has been assessed in transient replicon assays (Table 4).

Genotype 1a Y93H and Y93N substitutions each conferred approximately a 7-fold loss in susceptibility to pibrentasvir, consistent with their selection at 10-fold, but not at 100-fold, over the EC_50_. Generation of either the single amino acid substitution Q30D or the double substitution H58D+Y93H requires two nucleotide changes in the NS5A coding sequence. The higher genetic barrier to the generation of these substitutions is consistent with their low prevalence (only 1 colony each) in the resistance selection study. The Q30D and H58D+Y93H amino acid substitutions conferred 94- and 2,238-fold losses in susceptibility to pibrentasvir, respectively. Of note, genotype 1a H58D by itself does not confer resistance to pibrentasvir (Table 5), and Y93H alone confers a 6.7-fold loss in susceptibility to pibrentasvir (Table 4).

**TABLE 5:** Antiviral activity of pibrentasvir against HCV replicons of genotypes 1a and 1b containing NS5A with amino acid substitutions that confer resistance to other NS5A inhibitors

HCV genotype and NS5A amino acid substitution(s)	Pibrentasvir EC_50_^*a*^		Replication efficiency (%)^*b*^
	Mean ± SD (pM)	Fold change^*b*^	
1a-H77			
Wild type	0.72 ± 0.45		100
M28T	1.5 ± 1.1	2.1	89
M28V	1.3 ± 0.86	1.8	87
Q30E	1.7 ± 0.39	2.4	70
Q30H	0.74 ± 0.21	1.0	90
Q30R	1.2 ± 0.62	1.7	86
L31M	0.76 ± 0.11	1.1	141
L31V	0.96 ± 0.85	1.3	297
P32L	1.2 ± 0.43	1.7	19
H58D	0.80 ± 0.17	1.1	97
Y93C	1.2 ± 0.57	1.7	22
Y93H	4.8 ± 1.5	6.7	40
Y93N	5.1 ± 2.1	7.1	35
M28T+Q30R	1.2 ± 0.21	1.6	28
M28T+Y93C	2.2 ± 0.47	3.1	18
Q30L+Y93H	0.42 ± 0.09	0.6	27
Q30R+L31M	1.7 ± 0.34	2.4	46
Q30R+H58D	77 ± 2.1	108	46
Q30R+Y93C	2.8 ± 0.64	3.8	5.3
Q30R+Y93H	187 ± 110	260	11
L31M+Y93C	4.4 ± 0.55	6.1	28
L31V+Y93H	68 ± 36	94	73
M28T+Q30R+L31M	3.3 ± 0.41	4.6	44
Q30R+L31M+Y93C	30 ± 1.0	42	6.8
1b-Con-1			
Wild type	1.9 ± 0.80		100
L28T	1.7 ± 0.44	0.9	18
Y93H	1.1 ± 0.27	0.6	38
Y93N	1.2 ± 0.25	0.6	52
L31M+Y93H	1.3 ± 0.24	0.7	15
L31V+Y93H	1.7 ± 0.31	0.9	30
P58S+Y93H	1.5 ± 0.45	0.8	44

a As determined in transient-transfection assays.

b Fold change or replication efficiency is relative to the value of the respective wild-type replicon.

Genotype 2a colonies that survived treatment with pibrentasvir at 10-fold over the EC_50_ harbored the double amino acid substitution P29S+K30G or F28S+M31I (Table 4). Genotype 2a substitution F28S, P29S, K30G, or M31I alone either decreased viral replication efficiency substantially *in vitro* or did not impact susceptibility to pibrentasvir (Table 6 and unpublished data), whereas the rare double substitution P29S+K30G (one colony) or F28S+M31I (two colonies) conferred a 2.3-fold or 14,448-fold loss in susceptibility to pibrentasvir, respectively. All of the three genotype 3a colonies selected by pibrentasvir contained Y93H, which conferred a 2.3-fold loss in susceptibility to pibrentasvir.

**TABLE 6:** Antiviral activity of pibrentasvir against HCV replicons containing NS5A from genotypes 2 to 6 with amino acid substitutions that confer resistance to other NS5A inhibitors

HCV genotype and NS5A amino acid substitution(s)	Pibrentasvir EC_50_^*a*^	
	Mean ± SD (pM)	Fold change
2a		
Wild type^*b*^	0.99 ± 0.36	
T24A	1.3 ± 0.30	1.3
F28S	1.2 ± 0.17	1.2
2b		
Wild type^*c*^	1.2 ± 0.39	
L28F	0.94 ± 0.27	0.8
L31M	1.5 ± 0.33	1.2
L31V	0.64 ± 0.20	0.5
3a		
Wild type	0.65 ± 0.16	
M28T	1.1 ± 0.02	1.7
Y93H	1.5 ± 0.19	2.3
4a		
Wild type	0.78 ± 0.14	
L28V	0.85 ± 0.23	1.1
L30H	1.1 ± 0.51	1.3
5a		
Wild type	0.93 ± 0.20	
L28I	0.98 ± 0.14	1.1
L31F	1.9 ± 0.11	2.1
L31V	0.75 ± 0.24	0.8
6a		
Wild type^*d*^	1.0 ± 0.31	
L31V	1.0 ± 0.38	1.0
T58A	1.4 ± 0.45	1.4
T58N	1.8 ± 0.71	1.8

a As determined in transient-transfection assays. Fold change is relative to the value of the respective wild-type replicon.

b The 2a wild-type replicon has M31 in NS5A.

c The 2b wild-type replicon has L31 in NS5A.

d The 6a wild-type replicon has L28 in NS5A.

### Activity of pibrentasvir against HCV replicons containing NS5A of genotypes 1 to 6 with amino acid substitutions that confer resistance to other NS5A inhibitors.

To investigate if pibrentasvir is active against common amino acid substitutions known to confer resistance to other NS5A inhibitors, its activity was evaluated in transient replicon assays against a panel of substitutions observed in genotype 1-infected patients treated with the NS5A inhibitor daclatasvir, elbasvir, ledipasvir, ombitasvir, or velpatasvir (13, 14, 21, 22, 24, 27, 30, 31). This panel included resistance-associated single, double, or triple substitutions in NS5A at amino acid positions 28, 30, 31, 32, 58, and/or 93, which confer different levels of resistance (EC_50_s of 5 to 50,000 pM) to these NS5A inhibitors. Pibrentasvir retained full activity against all of the genotype 1a and 1b single-position NS5A substitutions tested, except Y93H and Y93N in genotype 1a, which conferred a ≤7-fold increase in EC_50_ to pibrentasvir (EC_50_s, ≤5.1 pM). Pibrentasvir demonstrated little loss in activity against most of the double or triple amino acid substitutions tested (Table 5). For the double or triple amino acid substitutions that conferred resistance to pibrentasvir, the EC_50_s were ≤187 pM.

Pibrentasvir was also tested against replicons of genotype 2 to 6 containing a panel of NS5A amino acid substitutions that have been reported to confer resistance to daclatasvir, elbasvir, ledipasvir, ombitasvir, and/or velpatasvir (13, 14, 21, 22, 24, 30, 31). This panel included substitutions at NS5A amino acid position 24, 28, 29, 30, 31, 58, or 93 (Table 6). Pibrentasvir was active against all of these substitutions in NS5A of genotypes 2 to 6, with EC_50_s of <2 pM. Of note, pibrentasvir retained its activity against common substitutions at amino acid position 31 in genotype 2, as evidenced by its activity against the wild-type genotype 2a and 2b replicons which had M31 and L31, respectively, as well as the L31M and L31V substitutions in genotype 2b (Table 6). Genotype 3a Y93H, which confers ≥200-fold resistance to other NS5A inhibitors (21, 22, 25, 28, 31), conferred a 2.3-fold change in susceptibility to pibrentasvir.

### Activity of pibrentasvir against HCV replicons containing amino acid substitutions that confer resistance to HCV protease or polymerase inhibitors.

The potential for cross-resistance of pibrentasvir with HCV inhibitors of different DAA classes was assessed using a transient-transfection replicon assay with replicons containing key resistance-associated amino acid substitutions for NS3/4A protease or NS5B polymerase inhibitors (Table 7). The NS3 substitutions R155K, D168A, and D168V in genotype 1a and R155K, A156T, and D168V in genotype 1b that confer resistance to different NS3/4A PIs, including telaprevir, boceprevir, simeprevir, asunaprevir, paritaprevir, and/or grazoprevir (14), remained fully susceptible to inhibition by pibrentasvir. Similarly, the NS5B polymerase resistance-associated substitutions C316Y, M414T, Y448C, Y448H, S556G, and S559G in genotype 1a and S282T, C316Y, Y448H, and S556G in genotype 1b did not confer any resistance to pibrentasvir. Among these NS5B amino acid substitutions, S282T is the key substitution associated with resistance to the nucleotide analog polymerase inhibitor sofosbuvir whereas the other substitutions are associated with resistance to the nonnucleoside polymerase inhibitor dasabuvir (32, 33).

**TABLE 7:** Antiviral activity of pibrentasvir against HCV replicons containing amino acid substitutions that confer resistance to NS3/4A protease or NS5B polymerase inhibitors

DAA target and HCV genotype	Amino acid substitution	Pibrentasvir EC_50_ (pM)^*a*^	Fold change in pibrentasvir EC_50_^*a*^
NS3			
1a	Wild type	0.94	
	R155K	0.72	0.76
	D168A	0.77	0.82
	D168V	0.79	0.84
1b	Wild type	2.7	
	R155K	1.4	0.54
	A156T	1.4	0.52
	D168V	2.5	0.91
NS5B			
1a	Wild type	1.3	
	C316Y	1.3	1.0
	M414T	1.6	1.2
	Y448C	1.0	0.77
	Y448H	1.0	0.77
	S556G	1.6	1.2
	S559G	0.6	0.46
1b	Wild type	1.8	
	S282T	3.1	1.7
	C316Y	1.8	1.0
	Y448H	1.9	1.1
	S556G	1.9	1.1

a As determined in transient-transfection assays. Fold change is relative to the value of the respective wild-type replicon.

### Combination studies of pibrentasvir with HCV inhibitors of other classes in HCV replicon cells.

The antiviral effects of the combination of pibrentasvir with HCV inhibitors of other classes were evaluated in genotype 1b HCV replicon cells. These HCV inhibitors included the next-generation HCV NS3/4A PI glecaprevir (EC_50_, 0.94 nM) (34), IFN-α (EC_50_, 1.2 IU/ml), and ribavirin (RBV) (EC_50_, 19 μM). The inhibitory effects produced by each compound alone and in combination at concentrations up to approximately 8-fold above or below their respective EC_50_s were compared using a checkerboard combination format. Based on MacSynergy II analysis, the combinations of pibrentasvir with these HCV inhibitors resulted in minor or moderate synergistic antiviral activity (Table 8).

**TABLE 8:** Antiviral activity of the combination of pibrentasvir with HCV inhibitors of other classes *in vitro*

HCV inhibitor^*a*^	Synergy vol (μM^2^%)	Antagonism vol (μM^2^%)	Interaction type
IFN-α	43 ± 4.7	−1.4 ± 0.1	Minor synergy
RBV	29 ± 7.4	−2.8 ± 0.3	Minor synergy
Glecaprevir (NS3/4A PI)	73 ± 17	−1.9 ± 0.4	Moderate synergy

a IFN-α, interferon alpha; RBV, ribavirin; PI, protease inhibitor.

## DISCUSSION

Pibrentasvir is a next-generation NS5A inhibitor with potent (EC_50_s, 1.4 to 5.0 pM) and pan-genotypic antiviral activity against HCV replicons containing NS5A from all major HCV genotypes. Pibrentasvir demonstrated similar *in vitro* antiviral activities against replicons with NS5A from a panel of clinical samples of genotypes 1 to 6, indicating that pibrentasvir can inhibit HCV with clinically relevant sequence diversity in NS5A. In addition, pibrentasvir retained its activity against common NS5A amino acid substitutions that confer resistance to other NS5A inhibitors. It also demonstrated a high genetic barrier to resistance, selecting a small number or no colonies with resistance-conferring amino acid substitutions in replicons containing NS5A from genotypes 1a, 1b, 2a, 2b, 3a, 4a, 5a, and 6a. Pibrentasvir was active against key resistance-associated amino acid substitutions for NS3/4A PIs or NS5B polymerase inhibitors. The combination of pibrentasvir with HCV inhibitors of other classes produced synergistic antiviral activity *in vitro*.

Pibrentasvir demonstrates improved pan-genotypic anti-HCV activity compared to all currently approved NS5A inhibitors. Ledipasvir has EC_50_s of 4 to 31 pM for genotype 1 but has reduced potency against other genotypes, especially for genotypes 2a and 3a (EC_50_s, ≥16,000 pM) (20). Genotype 2 with the common NS5A M31 polymorphism is less susceptible to daclatasvir than genotype 2 with NS5A L31 (genotype 2a with M31, EC_50_ of 6,700 pM; genotype 2b with M31, EC_50_ of 64,000 pM) (26). Genotype 2b with the NS5A M31 polymorphism is also less susceptible to elbasvir (EC_50_, 3,000 pM) than genotype 2b with L31 (23). The EC_50_s of ombitasvir against chimeric replicons containing NS5A from genotypes 1 to 5 range from 2 to 12 pM, and ombitasvir retains activity against the genotype 2 NS5A M31 polymorphism, but its EC_50_ against a genotype 6a NS5A chimeric replicon is 366 pM (22). The genotype 2 NS5A M31 polymorphism has no impact on the activity of pibrentasvir (genotype 2a with M31, EC_50_ of 2.3 pM). The recently approved NS5A inhibitor velpatasvir has an EC_50_ of 75 pM against genotype 5, while the EC_50_ values range from 6 to 15 pM against the other genotypes (21).

Pibrentasvir selected a small number of drug-resistant colonies in replicons containing NS5A from genotypes 1 to 6. Among the colonies selected by pibrentasvir, quite a number harbored substitutions resulting from two nucleotide changes (e.g., genotype 1a H58D+Y93H or Q30D, and genotype 2a P29S+K30G or F28S+M31I) and were present at very low prevalence (1 to 2 colonies out of 2 × 10^6^ input cells). This is consistent with the resistance data shown in Tables 5 and 6 that pibrentasvir maintained activity against common substitutions in NS5A of genotypes 1 to 6 resulting from single nucleotide changes but may have reduced activity against some of the double or triple amino acid substitutions, the generation of which requires overcoming a genetic barrier higher than that of a substitution resulting from a single nucleotide change.

Pibrentasvir has an improved resistance profile compared to the profiles of other NS5A inhibitors. Pibrentasvir maintains its potency *in vitro* against most of the resistance-associated substitutions detected in genotype 1-infected patients who have failed an NS5A inhibitor-containing regimen. Among the genotype 1 substitutions tested as shown in Table 5, the single-position NS5A substitutions, including M28T/V, Q30E/H/R, L31M/V, P32L, H58D, and Y93C/H/N, confer different levels of resistance to other NS5A inhibitors (13, 14, 20–22, 24, 30, 31), but all of them are susceptible to inhibition by pibrentasvir. Of note, genotype 1a Y93C, Y93H, and Y93N substitutions confer high levels of resistance to approved first-generation NS5A inhibitors (daclatasvir, ombitasvir, and ledipasvir), with EC_50_s ranging from 4,500 to 500,000 pM (22, 35, 36). Even for the newer NS5A inhibitors elbasvir and velpatasvir, Y93H and Y93N confer 220- to 2,800-fold resistance (27, 37). In contrast, Y93C confers no resistance to pibrentasvir, while Y93H and Y93N each confers only a 7-fold resistance, resulting in an EC_50_ of 5 pM. In HCV-infected patients who have failed an NS5A inhibitor-containing regimen, it is more common to detect viral variants with single-position NS5A substitutions than those with double or triple substitutions (22, 35, 36). However, if multiple-position substitutions are generated, they usually confer a high level of resistance (200- to >30,000-fold) to NS5A inhibitors (12, 22, 35–37). Although pibrentasvir has reduced activity against some of the genotype 1 double or triple substitutions seen clinically with other NS5A inhibitors, the impact of these substitutions on pibrentasvir EC_50_s (≤260-fold decrease) is less, in some cases substantially less, than the effects on the other NS5A inhibitors.

Pibrentasvir also retained its activity when tested against a panel of chimeric replicons containing NS5A from genotypes 2 to 6 with substitutions at amino acid position 24, 28, 30, 31, 58, or 93, which conferred resistance (20- to ≥100,000-fold) to ombitasvir, daclatasvir, and ledipasvir (22, 24, 25, 28). Some of these substitutions have also been reported to confer resistance to elbasvir and velpatasvir (21, 31). Among the non-genotype 1 substitutions tested, Y93H in genotype 3a confers a high level of resistance (20- to >2,500-fold) to other NS5A inhibitors (daclatasvir, ledipasvir, ombitasvir, elbasvir, and velpatasvir) (21, 22, 25, 28, 31) but has little impact on the potency of pibrentasvir (2.3-fold increase in EC_50_). In addition, the common genotype 2 polymorphism M31, which confers resistance to daclatasvir, ledipasvir, and elbasvir, as discussed above (23, 24, 26), has no impact on the activity of pibrentasvir.

The potent anti-HCV activity and high genetic barrier to development of drug resistance observed with pibrentasvir *in vitro* have translated into robust potency *in vivo*. Pibrentasvir has been evaluated in a 3-day dose-ranging monotherapy study in genotype 1-infected treatment-naive adults. The mean maximal decrease in HCV plasma RNA from the baseline at the end of the 3-day monotherapy was ≥4.1 log_10_ IU/ml for daily doses of 40 to 400 mg, with a 4.5-log_10_ IU/ml decrease for the 120-mg dose selected for phase 3 clinical studies (38). This viral load decline compares favorably with declines observed for monotherapy studies for other NS5A inhibitors (17–19, 39). Importantly, during this monotherapy study, NS5A amino acid substitutions emerged in only 3 out of the 40 (7.5%) patients, all of whom had received 120 or 400 mg of pibrentasvir (40). This low rate of emergence of amino acid substitutions during monotherapy is consistent with the high genetic barrier to resistance observed for pibrentasvir *in vitro*.

The combination of pibrentasvir with glecaprevir, a next-generation HCV NS3/4A PI, resulted in moderate synergy *in vitro*. Pibrentasvir has been coadministered with glecaprevir in clinical studies, and in SURVEYOR-I and SURVEYOR-II studies the optimized combination (300 mg of glecaprevir and 120 mg of pibrentasvir) achieved high SVR rates (modified intent-to-treat [mITT] SVR of ≥97%) in treatment-naive noncirrhotic patients with HCV genotype 1, 2, 3, 4, 5, or 6 infection (41–43). The combination of glecaprevir and pibrentasvir has also been studied in noncirrhotic genotype 1-infected patients who had failed a prior DAA-containing regimen in the MAGELLAN-I study (44). The majority of the patients had detectable resistance-associated amino acid variants (polymorphisms and/or substitutions) before the treatment with glecaprevir and pibrentasvir in the study, especially variants in NS5A, which have been reported to persist for years after treatment (35, 45, 46). Both glecaprevir and pibrentasvir have been shown *in vitro* to be active against HCV replicons with amino acid substitutions that confer resistance to first-generation NS3/4A PIs and NS5A inhibitors, respectively (34); thus, the combination of glecaprevir and pibrentasvir is expected to be efficacious for patients who are infected with HCV harboring substitutions that confer resistance to these DAAs. The combination of glecaprevir and pibrentasvir, indeed, demonstrated robust efficacy (mITT SVR of 96%) in HCV genotype 1-infected patients who had previously failed a DAA-containing regimen, regardless of the diverse profile and high prevalence of NS3 and/or NS5A resistance-associated polymorphisms/substitutions among these patients prior to the study (44).

In summary, the potent anti-HCV activity of pibrentasvir against replicons containing NS5A from genotypes 1 to 6 and the favorable pibrentasvir resistance profile *in vitro* have made pibrentasvir an excellent candidate to be combined with HCV inhibitors of other classes for the treatment of chronic HCV infection. Phase 2 clinical studies evaluating coadministration of glecaprevir and pibrentasvir have yielded robust SVR rates in treatment-naive noncirrhotic patients infected with HCV genotypes 1 to 6 (mITT SVR of ≥97%), as well as in genotype 1-infected patients who had failed a prior DAA-containing regimen (mITT SVR of 96%). These encouraging results provide the basis for the investigation of this combination regimen in larger cohorts of noncirrhotic and cirrhotic patients infected with HCV of genotypes 1 to 6, with or without a history of treatment with a DAA-containing regimen, in ongoing phase 3 clinical studies.

## MATERIALS AND METHODS

### Compounds.

Pibrentasvir, methyl {(2*S*,3*R*)-1-[(2*S*)-2-{5-[(2*R*,5*R*)-1-{3,5-difluoro-4-[4-(4-fluorophenyl)piperidin-1-yl]phenyl}-5-(6-fluoro-2-{(2*S*)-1-[*N*-(methoxycarbonyl)-*O*-methyl-l-threonyl]pyrrolidin-2-yl}-1*H*-benzimidazol-5-yl)pyrrolidin-2-yl]-6-fluoro-1*H*-benzimidazol-2-yl}pyrrolidin-1-yl]-3-methoxy-1-oxobutan-2-yl}carbamate (Fig. 1), and glecaprevir (ABT-493; identified by AbbVie and Enanta) were synthesized at AbbVie. IFN-α and ribavirin were purchased from Sigma-Aldrich (St. Louis, MO).

### Antiviral activity of pibrentasvir in HCV stable replicon cells.

Pibrentasvir was tested against nine stable HCV subgenomic replicon cell lines using a luciferase reporter assay as described previously (22). These include genotype 1a-H77 (GenBank accession number NC_004102), genotype 1b-Con1 (GenBank accession number AJ238799), genotype 2a-JFH-1 (GenBank accession number AB047639), and six chimeras of genotype 1b-Con1 containing the N-terminal region of NS5A from genotype 2a, 2b, 3a, 4a, 5a, or 6a (22). All replicon constructs were bicistronic subgenomic replicons similar to those described by Bartenschlager and coworkers, and the stable replicon cell lines were generated by introducing these constructs into the Huh-7 human hepatoma cell line (47). The inhibitory effect of pibrentasvir on HCV replication in replicon cells was determined in Dulbecco's modified Eagle's medium (DMEM) containing 5% fetal bovine serum with or without 40% human plasma (Bioreclamation, Westbury, NY). The cells were incubated with pibrentasvir for 3 days and were subsequently lysed and processed according to the manufacturer's instructions to measure luciferase reporter activity (luciferase assay system; Promega, Madison, WI) using a Victor II luminometer (Perkin-Elmer, Waltham, MA). The 50% effective concentration (EC_50_) value was calculated using nonlinear regression curve fitting to the four-parameter logistic equation in GraphPad Prism, version 4/5, software.

### Cytotoxicity of pibrentasvir.

The cytotoxicity of pibrentasvir was determined by an MTT [3-(4,5-dimethyl-2-thiazolyl)-2,5-diphenyl-2H-tetrazolium bromide] (Sigma-Aldrich, St. Louis, MO) colorimetric assay. Briefly, Huh-7 cells containing genotype 1a replicon or HepG2 cells (4,000 cells/well) were plated in 96-well plates and incubated overnight before the addition of pibrentasvir. For MT4 cells, 10,000 cells/well were plated immediately before the addition of pibrentasvir. Detection of cytotoxicity with MTT and calculation of the pibrentasvir 50% cytotoxicity concentration (CC_50_) value were performed as previously described (22).

### Activity of pibrentasvir against human immunodeficiency virus 1 and hepatitis B virus.

Antiviral activity assays for HIV-1 and HBV were performed at Southern Research Institute. Pibrentasvir was tested in an HIV-1 antiviral cytoprotection assay using CEM-SS cells and the IIIB strain of HIV-1. Briefly, virus and cells were mixed in the presence of pibrentasvir or zidovudine (AZT; positive control) and incubated for 6 days. The titers of the virus were determined beforehand such that the virus-infected control wells exhibited approximately 85% to 95% loss of cell viability due to virus replication. Therefore, antiviral effect or cytoprotection was observed when a compound prevented virus replication. Six days after infection, 20 to 25 μl of MTS [3-(4,5-dimethylthiazol-2-yl)-5-(3-carboxymethoxyphenyl)-2-(4-sulfophenyl)-2H-tetrazolium salt] reagent was added per well, and the microtiter plates were then incubated for 4 to 6 h to assess cell viability. Plates were read spectrophotometrically at 490/650 nm with a Molecular Devices Vmax or SpectraMax Plus plate reader.

The anti-HBV assay employed real-time quantitative PCR (qPCR; TaqMan) to directly measure extracellular HBV DNA copy number. Briefly, HepG2 2.2.15 cells were plated in 96-well microtiter plates. After 16 to 24 h the confluent monolayer of cells was washed, and the medium was replaced with complete medium containing serial dilutions of pibrentasvir or lamivudine (3TC; positive control). Three days later the culture medium was replaced with fresh medium containing the same compound. Six days following the initial administration of test compounds, the cell culture supernatant was collected and treated with pronase, and the HBV precore/core region was amplified in a real-time quantitative TaqMan qPCR assay. Antiviral activity expressed as the EC_50_ was calculated from the reduction in HBV DNA levels.

### Antiviral activity of pibrentasvir against HCV replicons containing NS5A from HCV-infected patients.

The activity of pibrentasvir against HCV NS5A from individual patients infected with HCV genotypes 1 to 6 was determined by testing the susceptibility of a panel of genotype 1b Con1 replicon-based shuttle vector constructs containing the respective NS5A genes. Descriptions of the replicon shuttle vectors and the transient replicon assay have been reported previously (22). For the analysis of patients' NS5A sequences, we define a polymorphism as an amino acid difference in a sequence of a baseline sample relative to the sequence of the appropriate reference sequence. Polymorphisms were assessed at the following amino acid positions that are associated with resistance to the NS5A inhibitor class: 24, 28, 29, 30, 31, 32, 58, 62, 92, and 93 for genotype 1a; 24, 28, 29, 30, 31, 32, 54, 58, 62, 92, and 93 for genotype 1b; 24, 28, 29, 30, 31, 32, 58, 92, and 93 for genotypes 2 to 6.

### Selection of amino acid substitutions conferring resistance to pibrentasvir in replicon cells.

Resistance selection was conducted with HCV stable replicon cells containing NS5A from genotype 1a-H77, 1b-Con1, 2a, 2b, 3a, 4a, 5a, or 6a as described previously (22). One million replicon cells were plated in 150-mm cell culture plates and grown in the presence of G418 (400 μg/ml) and pibrentasvir or ombitasvir (control) at a concentration that was either 10- or 100-fold above the EC_50_ for the respective replicon cell line. The medium was replaced with fresh medium containing G418 and pibrentasvir or ombitasvir every 3 to 4 days. After approximately 3 weeks of treatment, replicon cells that were cleared of replicon RNA were unable to survive in G418-containing medium while replicon cells containing drug-resistant amino acid substitutions survived and formed colonies. To characterize the HCV replicons present in the colonies that survived the drug selection, 20 colonies, or all of the surviving colonies if there were fewer than 20, were picked from each treatment condition and expanded for genotypic characterization. The expanded colonies were lysed in CellsDirect lysis buffer (Invitrogen, Waltham, MA), and the NS5A coding region from each colony was amplified by reverse transcription-PCR (RT-PCR) using gene-specific primers. For genotype 1a and 1b colonies, the full-length NS5A sequence was determined, whereas for colonies with a chimeric replicon containing the N-terminal region of NS5A from genotype 2a, 2b, 3a, 4a, 5a, or 6a, the sequence encoding the NS5A N-terminal region was determined. The amplified samples were sequenced using an ABI Prism dye terminator ready reaction cycle sequencing kit and were analyzed on an Applied Biosystems 3100 genetic analyzer. Following translation of DNA sequence to amino acid sequence, the HCV sequences were then compared with those of the corresponding untreated HCV replicon cells. We define a substitution in a replicon as an amino acid difference in a sequence from a posttreatment sample relative to the sequence of the corresponding untreated sample. For resistance selection studies, substitutions associated with resistance to the NS5A inhibitor class at the following amino acid positions were examined: 24, 28, 29, 30, 31, 32, 58, 62, 92, and 93 for genotype 1a; 24, 28, 29, 30, 31, 32, 54, 58, 62, 92, and 93 for genotype 1b; 24, 28, 29, 30, 31, 32, 58, 92, and 93 for genotypes 2 to 6. In addition to these listed positions, NS5A substitutions at other amino acid positions that were detected in more than one colony and conferred ≥2-fold increase in pibrentasvir EC_50_s are also reported.

### Effects of amino acid substitutions on the antiviral activity of pibrentasvir in HCV replicon cells.

HCV replicons were constructed with mutations encoding amino acid substitutions of interest in NS5A (genotypes 1 to 6) as well as those in NS3 or NS5B (genotype 1 only) to evaluate their susceptibility to pibrentasvir in transient replicon assays, as previously described (22). Mutagenesis was performed by using a Change-IT Multiple Mutation site-directed mutagenesis kit (USB) or by cloning a synthesized DNA fragment encoding the amino acid substitution(s). After mutagenesis was confirmed by sequence analysis, the plasmids were linearized and transcribed using a TranscriptAid T7 High Yield Transcription kit (Fermentas, Waltham, MA). Huh-7 cells were transfected with the replicon RNAs; inhibition of replication of these HCV replicons by pibrentasvir was measured using a luciferase assay. Replication efficiency was calculated as a percentage of wild-type replication as described previously (22).

### Combination of pibrentasvir with HCV inhibitors of other classes in HCV replicon cells.

The combination of pibrentasvir with HCV inhibitors of other classes was studied in genotype 1b-Con1 replicon cells to determine if the combinations produced synergistic, additive, or antagonistic antiviral effects. Each compound was serially diluted 2-fold, and 6 dilutions of pibrentasvir were combined with 6 dilutions of another HCV inhibitor according to the checkerboard method (48). The concentrations tested were chosen to ensure that the EC_50_ value of each compound was in the middle of the serial dilution range. The extent of replication of the HCV replicon was determined by a luciferase reporter assay. The resulting data were analyzed using the MacSynergy II program (48–50). The assignment of the potential importance of synergy or antagonism volumes was based on the guidelines provided in the MacSynergy II user's manual (49): >100 μM^2^%, strong synergy; 50 to 100 μM^2^%, moderate synergy; 25 to 50 μM^2^%, minor synergy; −25 to 25 μM^2^%, insignificant synergy/antagonism (additive); −50 to −25 μM^2^%, minor antagonism; −100 to −50 μM^2^%, moderate antagonism; <−100 μM^2^%, strong antagonism.

## References

[1] Messina JP, Humphreys I, Flaxman A, Brown A, Cooke GS, Pybus OG, Barnes E 2015 Global distribution and prevalence of hepatitis C virus genotypes. Hepatology 61:77–87. doi:10.1002/hep.27259. 25069599 PMC4303918

[2] Gower E, Estes C, Blach S, Razavi-Shearer K, Razavi H 2014 Global epidemiology and genotype distribution of the hepatitis C virus infection. J Hepatol 61(Suppl):S45–S57. doi:10.1016/j.jhep.2014.07.027. 25086286

[3] Smith DB, Bukh J, Kuiken C, Muerhoff AS, Rice CM, Stapleton JT, Simmonds P 2014 Expanded classification of hepatitis C virus into 7 genotypes and 67 subtypes: updated criteria and genotype assignment web resource. Hepatology 59:318–327. doi:10.1002/hep.26744. 24115039 PMC4063340

[4] Sievert W, Altraif I, Razavi HA, Abdo A, Ahmed EA, Alomair A, Amarapurkar D, Chen CH, Dou X, El Khayat H, Elshazly M, Esmat G, Guan R, Han KH, Koike K, Largen A, McCaughan G, Mogawer S, Monis A, Nawaz A, Piratvisuth T, Sanai FM, Sharara AI, Sibbel S, Sood A, Suh DJ, Wallace C, Young K, Negro F 2011 A systematic review of hepatitis C virus epidemiology in Asia, Australia and Egypt. Liver Int 31(Suppl 2):S61–S80. 10.1111/j.1478-3231.2011.02540.x 21651703

[5] Arzumanyan A, Reis HM, Feitelson MA 2013 Pathogenic mechanisms in HBV- and HCV-associated hepatocellular carcinoma. Nat Rev Cancer 13:123–135. doi:10.1038/nrc3449. 23344543

[6] van der Meer AJ, Veldt BJ, Feld JJ, Wedemeyer H, Dufour JF, Lammert F, Duarte-Rojo A, Heathcote EJ, Manns MP, Kuske L, Zeuzem S, Hofmann WP, de Knegt RJ, Hansen BE, Janssen HL 2012 Association between sustained virological response and all-cause mortality among patients with chronic hepatitis C and advanced hepatic fibrosis. JAMA 308:2584–2593. doi:10.1001/jama.2012.144878. 23268517

[7] Kwong AD 2014 The HCV revolution did not happen overnight. ACS Med Chem Lett 5:214–220. doi:10.1021/ml500070q. 24672647 PMC3963459

[8] Asselah T, Boyer N, Saadoun D, Martinot-Peignoux M, Marcellin P 2016 Direct-acting antivirals for the treatment of hepatitis C virus infection: optimizing current IFN-free treatment and future perspectives. Liver Int 36(Suppl 1):47–57. doi:10.1111/liv.13027. 26725897

[9] Pawlotsky JM 2014 New hepatitis C therapies: the toolbox, strategies, and challenges. Gastroenterology 146:1176–1192. doi:10.1053/j.gastro.2014.03.003. 24631495

[10] Merck & Co., Inc. 2016 Zepatier (elbasvir and grazoprevir) tablets, for oral use, package insert. Merck & Co., Inc., Whitehouse Station, NJ https://www.merck.com/product/usa/pi_circulars/z/zepatier/zepatier_pi.pdf.

[11] Jacobson IM, Asante-Appiah E, Wong P, Black T, Howe A, Wahl J, Robertson MN, Nguyen B-Y, Shaughnessy M, Hwang P, Barr E, Hazuda D 2015 Prevalence and impact of baseline NS5A resistance-associated variants (RAVs) on the efficacy of elbasvir/grazoprevir (EBR/GZR) against GT1a infection. Hepatology 62(Suppl 1):1393A–1394A.

[12] McPhee F, Hernandez D, Yu F, Ueland J, Monikowski A, Carifa A, Falk P, Wang C, Fridell R, Eley T, Zhou N, Gardiner D 2013 Resistance analysis of hepatitis C virus genotype 1 prior treatment null responders receiving daclatasvir and asunaprevir. Hepatology 58:902–911. doi:10.1002/hep.26388. 23504694

[13] Nakamoto S, Kanda T, Wu S, Shirasawa H, Yokosuka O 2014 Hepatitis C virus NS5A inhibitors and drug resistance mutations. World J Gastroenterol 20:2902–2912. doi:10.3748/wjg.v20.i11.2902. 24659881 PMC3961994

[14] Lontok E, Harrington P, Howe A, Kieffer T, Lennerstrand J, Lenz O, McPhee F, Mo H, Parkin N, Pilot-Matias T, Miller V 2015 Hepatitis C virus drug resistance-associated substitutions: state of the art summary. Hepatology 62:1623–1632. doi:10.1002/hep.27934. 26095927

[15] Macdonald A, Harris M 2004 Hepatitis C virus NS5A: tales of a promiscuous protein. J Gen Virol 85:2485–2502. doi:10.1099/vir.0.80204-0. 15302943

[16] Masaki T, Suzuki R, Murakami K, Aizaki H, Ishii K, Murayama A, Date T, Matsuura Y, Miyamura T, Wakita T, Suzuki T 2008 Interaction of hepatitis C virus nonstructural protein 5A with core protein is critical for the production of infectious virus particles. J Virol 82:7964–7976. doi:10.1128/JVI.00826-08. 18524832 PMC2519576

[17] Nettles RE, Gao M, Bifano M, Chung E, Persson A, Marbury TC, Goldwater R, DeMicco MP, Rodriguez-Torres M, Vutikullird A, Fuentes E, Lawitz E, Lopez-Talavera JC, Grasela DM 2011 Multiple ascending dose study of BMS-790052, a nonstructural protein 5A replication complex inhibitor, in patients infected with hepatitis C virus genotype 1. Hepatology 54:1956–1965. doi:10.1002/hep.24609. 21837752

[18] Lawitz EJ, Gruener D, Hill JM, Marbury T, Moorehead L, Mathias A, Cheng G, Link JO, Wong KA, Mo H, McHutchison JG, Brainard DM 2012 A phase 1, randomized, placebo-controlled, 3-day, dose-ranging study of GS-5885, an NS5A inhibitor, in patients with genotype 1 hepatitis C. J Hepatol 57:24–31. doi:10.1016/j.jhep.2011.12.029. 22314425

[19] Lawitz J, Marbury T, Campbell A, Dumas E, Pilot-Matias T, Krishnan P, Setze C, Xie W, Podsadecki T, Bernstein B, Williams L 2012 Safety and antiviral activity of ABT-267, a novel NS5A inhibitor, during 3-day monotherapy: first study in HCV genotype-1 (GT1)-infected treatment-naive subjects. J Hepatol 56(Suppl 2):S469–S470. doi:10.1016/S0168-8278(12)61198-2.

[20] Cheng G, Tian Y, Doehle B, Peng B, Corsa A, Lee YJ, Gong R, Yu M, Han B, Xu S, Dvory-Sobol H, Perron M, Xu Y, Mo H, Pagratis N, Link JO, Delaney W 2016 In vitro antiviral activity and resistance profile characterization of the hepatitis C virus NS5A inhibitor ledipasvir. Antimicrob Agents Chemother 60:1847–1853. doi:10.1128/AAC.02524-15. 26824950 PMC4775926

[21] Cheng G, Yu M, Peng B, Lee YJ, Trejo-Martin A, Gong R, Bush C, Worth A, Nash M, Chan K, Yang H, Beran R, Tian Y, Perry J, Taylor J, Yang C, Paulson M, Delaney W, Link JO 2013 GS-5816, a second-generation HCV NS5A inhibitor with potent antiviral activity, broad genotypic coverage, and a high resistance barrier. J Hepatol 58(Suppl 1):S484–S485. doi:10.1016/S0168-8278(13)61192-7.

[22] Krishnan P, Beyer J, Mistry N, Koev G, Reisch T, DeGoey D, Kati W, Campbell A, Williams L, Xie W, Setze C, Molla A, Collins C, Pilot-Matias T 2015 *In vitro* and *in vivo* antiviral activity and resistance profile of ombitasvir, an inhibitor of hepatitis C virus NS5A. Antimicrob Agents Chemother 59:979–987. doi:10.1128/AAC.04226-14. 25451055 PMC4335823

[23] Liu R, Kong R, Mann P, Ingravallo P, Zhai Y, Xia E, Ludmerer S, Kozlowski J, Coburn C 2012 In vitro resistance analysis of Merck's HCV NS5a inhibitor MK-8742 demonstrates increased potency against clinical resistance variants and improved resistance profile. J Hepatol 56(Suppl 2):S334–S335. doi:10.1016/S0168-8278(12)60870-8.

[24] Wang C, Jia L, O'Boyle DR II, Sun JH, Rigat K, Valera L, Nower P, Huang X, Kienzle B, Roberts S, Gao M, Fridell RA 2014 Comparison of daclatasvir resistance barriers on NS5A from hepatitis C virus genotypes 1 to 6: implications for cross-genotype activity. Antimicrob Agents Chemother 58:5155–5163. doi:10.1128/AAC.02788-14. 24936600 PMC4135806

[25] Wang C, Valera L, Jia L, Kirk MJ, Gao M, Fridell RA 2013 In vitro activity of daclatasvir on hepatitis C virus genotype 3 NS5A. Antimicrob Agents Chemother 57:611–613. doi:10.1128/AAC.01874-12. 23089758 PMC3535966

[26] Zhou N, Han Z, Hartman-Neumann S, DeGray B, Ueland J, Vellucci V, Hernandez D, McPhee F 2016 Characterization of NS5A polymorphisms and their impact on response rates in patients with HCV genotype 2 treated with daclatasvir-based regimens. J Antimicrob Chemother 71:3495–3505. doi:10.1093/jac/dkw336. 27605597

[27] Doehle B, Dvory-Sobol H, Hebner C, Gontcharova V, Chodavarapu K, Ouyang W, Rodriguez-Torres M, Lawitz E, Yang C, McNally J, Link J, Mo H 2013 Deep sequencing of HCV NS5A from a 3-day study of GS-5816 monotherapy confirms the potency of GS-5816 against pre-existing genotype 1-3 NS5A resistance-associated variants abstr 470. Abstr 64th Annu Meet Am Assoc Study Liver Dis, Washington, DC.

[28] Hernandez D, Zhou N, Ueland J, Monikowski A, McPhee F 2013 Natural prevalence of NS5A polymorphisms in subjects infected with hepatitis C virus genotype 3 and their effects on the antiviral activity of NS5A inhibitors. J Clin Virol 57:13–18. doi:10.1016/j.jcv.2012.12.020. 23384816

[29] Gane E, Nahass R, Luketic V, Hwang P, Robertson M, Wahl J, Barr E, Haber B 2015 Efficacy of 12 or 18 weeks of grazoprevir plus elbasvir with ribavirin in treatment-naive, noncirrhotic HCV genotype 3-infected patients. J Hepatol 62(Suppl 2):S621. doi:10.1016/S0168-8278(15)30979-X. 28470815

[30] Gao M 2013 Antiviral activity and resistance of HCV NS5A replication complex inhibitors. Curr Opin Virol 3:514–520. doi:10.1016/j.coviro.2013.06.014. 23896281

[31] Liu R, Curry S, McMonagle P, Yeh WW, Ludmerer SW, Jumes PA, Marshall WL, Kong S, Ingravallo P, Black S, Pak I, DiNubile MJ, Howe AY 2015 Susceptibilities of genotype 1a, 1b, and 3 hepatitis C virus variants to the NS5A inhibitor elbasvir. Antimicrob Agents Chemother 59:6922–6929. doi:10.1128/AAC.01390-15. 26303801 PMC4604396

[32] Kati W, Koev G, Irvin M, Beyer J, Liu Y, Krishnan P, Reisch T, Mondal R, Wagner R, Molla A, Maring C, Collins C 2015 In vitro activity and resistance profile of dasabuvir, a nonnucleoside hepatitis C virus polymerase inhibitor. Antimicrob Agents Chemother 59:1505–1511. doi:10.1128/AAC.04619-14. 25534735 PMC4325770

[33] Lam AM, Espiritu C, Bansal S, Micolochick Steuer HM, Niu C, Zennou V, Keilman M, Zhu Y, Lan S, Otto MJ, Furman PA 2012 Genotype and subtype profiling of PSI-7977 as a nucleotide inhibitor of hepatitis C virus. Antimicrob Agents Chemother 56:3359–3368. doi:10.1128/AAC.00054-12. 22430955 PMC3370800

[34] Ng TI, Reisch T, Middleton T, McDaniel K, Kempf D, Lu L, Wang G, Jiang L, Or YS, Pilot-Matias T 2014 ABT-493, a potent HCV NS3/4A protease inhibitor with broad genotype coverage, abstr 636. Abstr 21st Annu Conf Retroviruses Opportunistic Infect, Boston, MA.

[35] Wang C, Sun JH, O'Boyle DR II, Nower P, Valera L, Roberts S, Fridell RA, Gao M 2013 Persistence of resistant variants in hepatitis C virus-infected patients treated with the NS5A replication complex inhibitor daclatasvir. Antimicrob Agents Chemother 57:2054–2065. doi:10.1128/AAC.02494-12. 23403428 PMC3632915

[36] Wong KA, Worth A, Martin R, Svarovskaia E, Brainard DM, Lawitz E, Miller MD, Mo H 2013 Characterization of hepatitis C virus resistance from a multiple-dose clinical trial of the novel NS5A inhibitor GS-5885. Antimicrob Agents Chemother 57:6333–6340. doi:10.1128/AAC.02193-12. 23877691 PMC3837913

[37] Black S, Pak I, Ingravallo P, McMonagle P, Chase R, Shaughnessy M, Hwang P, Haber B, Harrigan PR, Brumme C, Hazuda D, Howe AY 2015 Resistance analysis of virologic failures in hepatitis C genotype 1-infected patients treated with grazoprevir + elbasvir ± ribavirin: the C-WORTHY study, abstr P0891. Abstr 50th Annu Meet Eur Assoc Study Liver, Vienna, Austria.

[38] Lawitz EJ, O'Riordan WD, Asatryan A, Freilich BL, Box TD, Overcash JS, Lovell S, Ng TI, Liu W, Campbell A, Lin CW, Yao B, Kort J 2016 Potent antiviral activities of the direct-acting antivirals ABT-493 and ABT-530 with three-day monotherapy for hepatitis C virus genotype 1 infection. Antimicrob Agents Chemother 60:1546–1555. doi:10.1128/AAC.02264-15. PMC4775945 26711747

[39] Lawitz E, Freilich B, Link J, German P, Mo H, Han L, Brainard DM, McNally J, Marbury T, Rodriguez-Torres M 2015 A phase 1, randomized, dose-ranging study of GS-5816, a once-daily NS5A inhibitor, in patients with genotype 1-4 hepatitis C virus. J Viral Hepat 22:1011–1019. doi:10.1111/jvh.12435. 26183611

[40] Ng T, Pilot-Matias T, Tripathi R, Schnell G, Reisch T, Beyer J, Dekhtyar T, Asatryan A, Mensa FJ, Campbell AL, Kort J, Collin C 2015 Analysis of HCV genotype 1 variants detected during monotherapy and combination therapy with next generation HCV direct-acting antiviral agents ABT-493 and ABT-530. Hepatology 62(Suppl S1):558A.25716872

[41] Gane E, Lalezari J, Asatryan A, Greenbloom S, Hassanein TI, Ng T, Liu R, Lin C-W, Kort J, Mensa F 2016 100% SVR4 and favorable safety of ABT-493 + ABT-530 administered for 12 weeks in non-cirrhotic patients with genotypes 4, 5, or 6 infection (Surveyor-I). Gastroenterology 150(Suppl 1):S1047. doi:10.1016/S0016-5085(16)33540-5.

[42] Gane E, Poordad F, Wang S, Asatryan A, Kwo PY, Lalezari J, Wyles DL, Hassanein T, Aguilar H, Maliakkal B, Liu R, Lin CW, Ng TI, Kort J, Mensa FJ 2016 High efficacy of ABT-493 and ABT-530 treatment in patients with HCV genotype 1 or 3 infection and compensated cirrhosis. Gastroenterology 151:651–659.e651. doi:10.1053/j.gastro.2016.07.020. 27456384

[43] Poordad F, Felizarta F, Wang S, Asatryan A, Hassanein TI, Aguilar H, Lalezari J, Overcash JS, Ng T, Lovell SS, Lin C-W, Kort J, Mensa F 2016 High SVR rates with the combination of ABT-493 + ABT-530 for 8 weeks in non-cirrhotic patients with HCV genotype 1 or 2 infection. Gastroenterology 150(Suppl 1):S1046–S1047. doi:10.1016/S0016-5085(16)33537-5.

[44] Poordad F, Gordon SC, Asatryan A, Felizarta F, Reindollar RW, Landis C, Fried MW, Bernstein DE, Ng TI, Lin C, Liu R, Kort J, Mensa FJ 2016 High efficacy of ABT-493 and ABT-530 in HCV genotype 1 infected patients who have failed direct-acting antiviral-containing regimens: the Magellan-I study, abstr GS-11. Abstr 51st Annu Meet Eur Assoc Study Liver, Barcelona, Spain.

[45] Dvory-Sobol H, Wyles D, Ouyang W, Chodavarapu K, McNally J, Cheng W, Shafran S, Mangia A, Schwabe C, Miller MD, Mo H 2015 Long-term persistence of HCV NS5A variants after treatment with NS5A inhibitor ledipasvir. J Hepatol 62(Suppl 2):S221. doi:10.1016/S0168-8278(15)30073-8.

[46] Krishnan P, Tripathi R, Schnell G, Reisch T, Beyer J, Irvin M, Xie W, Larsen L, Cohen D, Podsadecki T, Pilot-Matias T, Collins C 2015 Resistance analysis of baseline and treatment-emergent variants in hepatitis C virus genotype 1 in the AVIATOR study with paritaprevir-ritonavir, ombitasvir, and dasabuvir. Antimicrob Agents Chemother 59:5445–5454. doi:10.1128/AAC.00998-15. 26100711 PMC4538512

[47] Lohmann V, Korner F, Koch J, Herian U, Theilmann L, Bartenschlager R 1999 Replication of subgenomic hepatitis C virus RNAs in a hepatoma cell line. Science 285:110–113. doi:10.1126/science.285.5424.110. 10390360

[48] Prichard MN, Shipman C Jr 1996 Analysis of combinations of antiviral drugs and design of effective multidrug therapies. Antivir Ther 1:9–20. 11322261

[49] Prichard MN, Aseltine KR, Shipman C Jr 1993 MacSynergy II, version 1.0. University of Michigan, Ann Arbor, MI.

[50] Prichard MN, Prichard LE, Shipman C Jr 1993 Strategic design and three-dimensional analysis of antiviral drug combinations. Antimicrob Agents Chemother 37:540–545. doi:10.1128/AAC.37.3.540. 8384816 PMC187704

